# Disparities in type 2 diabetes incidence trends during the COVID-19 pandemic in Sweden: a register-based population study

**DOI:** 10.1186/s12889-026-30127-z

**Published:** 2026-10-28

**Authors:** Josefine Andreasson, Dominik Dietler, Jonas Björk, Louise Bennet

**Affiliations:** 1 Division of Occupational and Environmental Medicine, Lund University, Lund, Sweden; 2 Clinical Studies Sweden, Forum South, Skåne University Hospital, Lund, Sweden; 3 Department of Clinical Sciences in Malmö, Lund University, Malmö, Sweden

**Keywords:** COVID-19, Type 2 diabetes, Epidemiology, Disparities, Pandemic, Disease management

## Abstract

**Background:**

The COVID-19 pandemic profoundly affected health care provision for chronic diseases, including type 2 diabetes. However, its effects on type 2 diabetes incidence remain poorly understood, particularly how they differ between population groups, which has not yet been examined in a Swedish context. We aimed to investigate trends in type 2 diabetes incidence in Sweden from 2016 to 2022, and to explore whether potential changes in type 2 diabetes incidence associated with the COVID-19 pandemic varied by sex, age, or migration status.

**Methods:**

Quarterly incidence rates of new-onset type 2 diabetes were calculated from individual-level register data for a dynamic population consisting of all adults (age ≥ 18 years) in Sweden during 2016–2019 (pre-pandemic) and 2020–2022 (pandemic). Incidence rate ratios (IRRs) for each pandemic year were modelled in multivariable Poisson regression analysis using the pre-pandemic years as reference, adjusted for age, sex, migration status, season, and long-term annual trend. Stratified and interaction analyses were performed based on age, sex, and migration status.

**Results:**

Overall, the incidence of type 2 diabetes showed an increasing underlying linear trend of 3.6% (IRR 1.036, 95% CI 1.028, 1.045) between 2016 and 2022. During the pandemic (2020–2022), the overall type 2 diabetes incidence decreased by 6% relative to the underlying linear trend (IRR 0.94, 95% CI 0.91, 0.97). The decrease was strongest in 2020 (IRR 0.90, 95% CI 0.87, 0.94) compared to 2021 (IRR 1.03, 95% CI 0.99, 1.06) and 2022 (IRR 0.95, 95% CI 0.91, 0.99). The decline in type 2 diabetes incidence in 2020 was most pronounced among individuals 65 years and older and in the native-born Swedish population.

**Conclusions:**

During the first year of the COVID-19 pandemic in Sweden, a decline in incidence of type 2 diabetes was observed that varied in magnitude by sex, age, and migration status. This finding may reflect differential but transient changes in health-seeking behaviour during the initial phases of the pandemic. Our results underscore the challenges of maintaining continuous health care for non-communicable diseases during a major health crisis in society, highlighting the need to ensure equitable access for vulnerable groups such as older persons.

**Supplementary Information:**

The online version contains supplementary material available at https://doi.org/10.1186/s12889-026-30127-z.

## Background

At least 11.1% of the global population are living with diabetes of which more than 90% have type 2 diabetes [1]. Type 2 diabetes is a severe chronic disease contributing to an increased risk of cardiovascular morbidity and mortality [2]. It furthermore increases the risk of diabetic complications including retinopathy, chronic kidney disease, and neurological complications [3], as well as cancers [4]. The global increase of type 2 diabetes is closely linked to the rising prevalence in obesity, driven by unhealthy eating habits, sedentary lifestyle, and socioeconomic vulnerability. These environmental factors, in interplay with genetic predisposition, significantly contribute to the risk of developing type 2 diabetes [2].

The COVID-19 pandemic, caused by the severe acute respiratory syndrome coronavirus 2 (SARS-CoV-2), had a profound impact on public health globally [5]. Beyond its direct impact on mortality and severe morbidity, the pandemic also indirectly affected mental health and lifestyles through public health restrictions and lockdown [6, 7]. Preventive measures such as remote work and social distancing have been reported to have negatively impacted physical activity levels and healthy eating habits [8], which can increase the risk for developing type 2 diabetes through reduced insulin sensitivity, increased visceral fat content, and impaired glycaemic control [2]. Furthermore, the pandemic substantially disrupted the provision of care for several chronic conditions. Routine diabetes follow-up visits were frequently postponed, adversely affecting diabetes care, particularly in primary care settings [9]. In addition, many individuals deferred or avoided seeking healthcare for non-COVID-19 conditions because of concerns about infection risk [10]. Together, these disruptions may have reduced adherence to chronic disease management protocols, resulting in less proactive management of individuals at high risk of type 2 diabetes and delays in diagnosis.

Studies on type 2 diabetes incidence in relation to the COVID-19 pandemic have shown inconsistent results. For instance, a study from Italy showed a significantly higher incidence of type 2 diabetes during the pandemic compared to the pre-pandemic period [11]. Data from England indicated a return to pre-pandemic trends in type 2 diabetes in 2022 after initial increase during the first two years of the pandemic [12], while a German study showed no change in type 2 diabetes incidence during the first two pandemic years compared to the pre-pandemic period [13]. Furthermore, some studies have suggested new-onset type 2 diabetes as an outcome following SARS-CoV-2 infection [14, 15], with risk of new-onset type 2 diabetes increasing with SARS-CoV-2 disease severity [16]. However, in our recent study [17], we found no evidence that SARS-CoV-2 infection was associated with incident type 2 diabetes. The observed increase in diabetes risk followed patterns similar to those of negative control outcomes unrelated to SARS-CoV-2 infection, suggesting that the apparent excess risk was largely attributable to increased detection among individuals hospitalized with COVID-19 rather than a direct effect of the infection itself.

In addition to sex and age, migration status is a significant factor for the risk of developing type 2 diabetes, with individuals with non-Western origin developing type 2 diabetes earlier in life and having twice the prevalence of type 2 diabetes compared to those without a migration background [18, 19], presumably due to high heritability, thrifty gene hypothesis, and gene environment interactions [20]. Early during the COVID-19 pandemic, it became evident that individuals with non-Western origin faced a higher risk of severe illness from SARS-CoV-2 infection [21, 22]. This raises important questions about potential disparities in the impact of the COVID-19 pandemic on type 2 diabetes incidence across migration groups. Identifying contributors to type 2 diabetes is critical for preventing the disease, reducing its burden and associated healthcare costs, and ultimately improving quality of life and promoting health equity.

Sweden adopted a relatively non-restrictive public health response mainly based on voluntary measures, such as recommendations on social distancing and working from home [23], rather than implementing a strict lockdown as in several other countries. At the same time, the country has a large immigrant population with 22% being born abroad and an additional 10% second-generation immigrants with one or both parents born abroad [24]. Sweden therefore provides an interesting setting for studying trends in type 2 diabetes across population groups. Hence, the purpose of this study was to investigate changes in overall trends in the incidence of type 2 diabetes in Sweden during the pre-pandemic and pandemic period, from 2016 to 2022, using nationwide register data for the entire adult population. Additionally, we sought to explore whether the changes in type 2 diabetes incidence associated with the COVID-19 pandemic varied by sex, age, or migration status.

## Methods

### Data sources

Data were obtained from multiple national registers with complete population coverage using the personal identification number as linkage [25]. All data were pseudonymized before disclosure. The Total Population Register from Statistics Sweden [26] provided demographic information, including immigration, emigration, and death. Data on diabetes diagnosis and prescription of anti-diabetic medication were retrieved from The Swedish National Diabetes Register (NDR) [27], a nationwide clinical quality register; the National Patient Register, which includes diagnoses from all inpatient stays and specialized outpatient care [29]; and the National Prescribed Drug Register [30]. Information on previous diabetes was available for 2005–2023 for inpatient care, outpatient care, and prescribed drugs. The registers providing data on diabetes are further described in Supplementary Methods 1.

### Study design and population

This dynamic population study was based on individual-level register data for all adults (aged ≥ 18 years) registered in Sweden anytime between 2016 and 2022. Individuals with any diabetes diagnosis (ICD-10 codes E08-E14) or prescribed diabetes medication (Anatomical Therapeutic Chemical [ATC] codes A10A/A10B) before 1 January 2016 were excluded. Individuals who met inclusion criteria contributed person-time starting on 1 January 2016, 1 January of the year in which the individual turned 18, or one year after immigration to Sweden. The one-year period after immigration was chosen to allow registration of pre-existing diabetes cases in the Swedish healthcare registers and hence, avoid misclassification as new cases. Individuals were followed until the date of new-onset type 2 diabetes (ICD-10 codes E11/E14 or prescription of drugs with ATC codes A10A/A10B), the earliest occurrence of other diabetes (ICD-10 codes E08-E10/E12/E13), death, emigration, or end of follow-up (31 December 2022).

### Type 2 diabetes onset

**Fig. 1:**
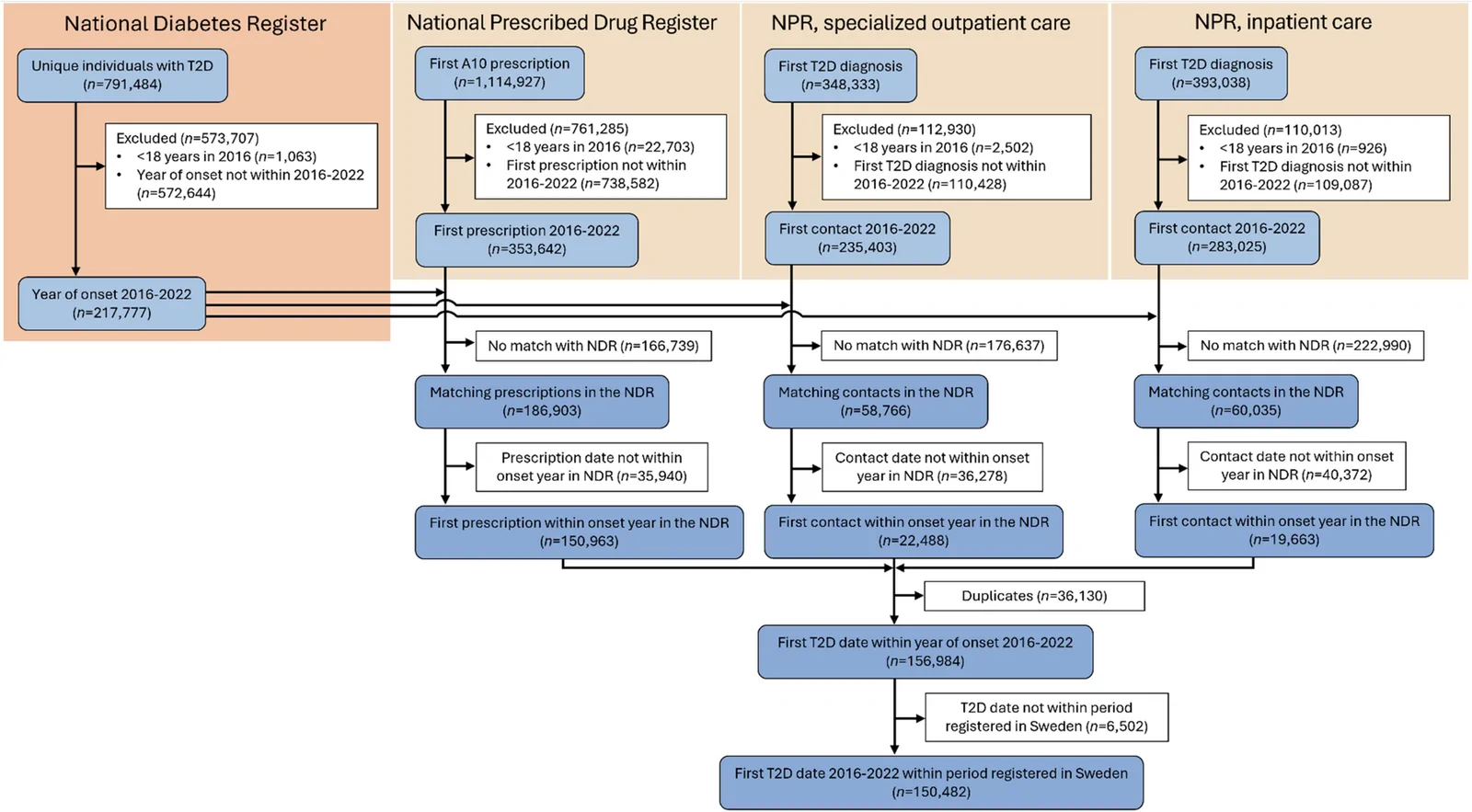
Data extraction of type 2 diabetes onset date from national registers to identify quarterly new-onset type 2 diabetes within the study period 2016–2022. NDR = National Diabetes Register; NPR = National Patient Register; T2D = type 2 diabetes

The outcome of interest was new-onset type 2 diabetes. We triangulated data from the NDR, the National Prescribed Drug Register, and the National Patient Register to derive the date of type 2 diabetes onset (Fig. 1). The NDR served as the primary standard as a clinically validated register of diagnosed diabetes, however, it records only the year of onset, and the registration date does not always reflect the true timing of diagnosis. The exact date of onset, required for seasonal adjustment, was therefore reconstructed from the National Patient Register and the National Prescribed Drug Register.

In the National Patient Register, ICD-10 codes E11 (type 2 diabetes) and E14 (unspecified diabetes) were used to identify type 2 diabetes diagnosis date. In the National Prescribed Drug Register, the earliest dispensation date of anti-diabetic medication (ATC codes A10A/A10B: anti-diabetic drugs, including insulins and glucose-lowering medications) served as potential onset date of type 2 diabetes. To reduce the risk of misclassification from any single source, an individual was considered a new type 2 diabetes case if they were registered in the NDR and had either a clinical diagnosis of type 2 diabetes or first prescription of anti-diabetic medication during the year of onset registered in NDR. The date of type 2 diabetes onset was defined as the earliest date between type 2 diabetes diagnosis and anti-diabetic medication prescription.

### Covariates

Age was categorized into 5-year age groups (18–24, 25–29, …, 95–99, 100 or older) for calculating incidence. Here, migration status refers to a combined classification of country of birth and parental origin. Although the term ‘migration status’ is used in this study for consistency, it includes both individuals who have migrated themselves (first-generation immigrants with either Western or non-Western origin), individuals born in Sweden to one or two foreign-born parents (second-generation immigrants), and individuals born in Sweden to two Swedish-born parents (native-born). Non-Western origin among first-generation migrants was defined as being born in a country in the Middle East, Africa, Asia, South America, former Soviet Union, or if registered as nationless. Western origin was defined as being born in the remaining regions except Sweden [19].

### Statistical analysis

#### Type 2 diabetes incidence trends 2016–2022

The incidence for new-onset type 2 diabetes was calculated for each quarter during the study period. The total number of cases and person-years at risk were aggregated from the three months in each quarter. The incidence for the current quarter was derived from the pooled cases and person-years over this interval. Quarterly incidence rate (IR) was reported per 100,000 person-years for the total population with 95% CIs, and stratified by age, sex, and migration status.

To account for differences in age and sex distribution when presenting incidence stratified by migration status, direct age and sex standardization was performed using the adult population (aged ≥ 18 years) residing in Sweden on 1 January 2016 as reference population. The population was divided into 5-year age groups and stratified by sex. Age- and sex-specific weights were calculated based on the proportion of individuals in each group relative to the total population (see Supplementary Methods 2).

#### Impact of COVID-19 pandemic on type 2 diabetes incidence

To assess deviation from the underlying long-term trend during the pandemic years, annual type 2 diabetes incidence rate ratios (IRRs) for the years 2020, 2021, and 2022 were modelled with the pre-pandemic period 2016–2019 as reference using generalized linear models with a log link function and Poisson distribution with robust standard errors to account for overdispersion, with logarithmic person-years at risk included as an offset. Covariates in the model included 5-year age group, sex, and migration status, together with quarter (Q1-Q4) to account for seasonal variation, and a continuous calendar year term to account for long-term annual linear trend in incidence. The four oldest age categories were collapsed into a single category (≥85 years) due to small sample size. Results were presented as IRR and 95% CIs.

#### Effect modification

To assess effect modification, we tested for statistical interaction between pandemic year and each of the covariates age group (18–64 years vs. ≥65 years), sex, and migration status. For each category, we fit a separate model using the same methodology as described above which additionally included an interaction term between the pandemic year and age, sex, and migration status, respectively. We then compared the model with and without the interaction term using likelihood ratio tests (LRT) to evaluate whether inclusion of interaction term significantly improved the model fit (*p* < 0.05). All data analyses were performed using R Statistical Software version 4.5.0 [31].

#### Sensitivity analysis

In separate analyses, we defined type 2 diabetes onset as the year of onset registered in the NDR, regardless of additional supporting evidence from other registers. This less restrictive definition of type 2 diabetes onset was used to allow for inclusion of those cases potentially not documented in clinical encounters or pharmacy data, but that nonetheless were recorded in NDR. The additional cases identified using this definition were likely individuals diagnosed in primary care, where the majority of type 2 diabetes cases are managed [32]. As information on month of onset was not available in NDR, regression models in sensitivity analyses did not include quarter.

## Results

### Overall trends in type 2 diabetes incidence

**Fig. 2:**
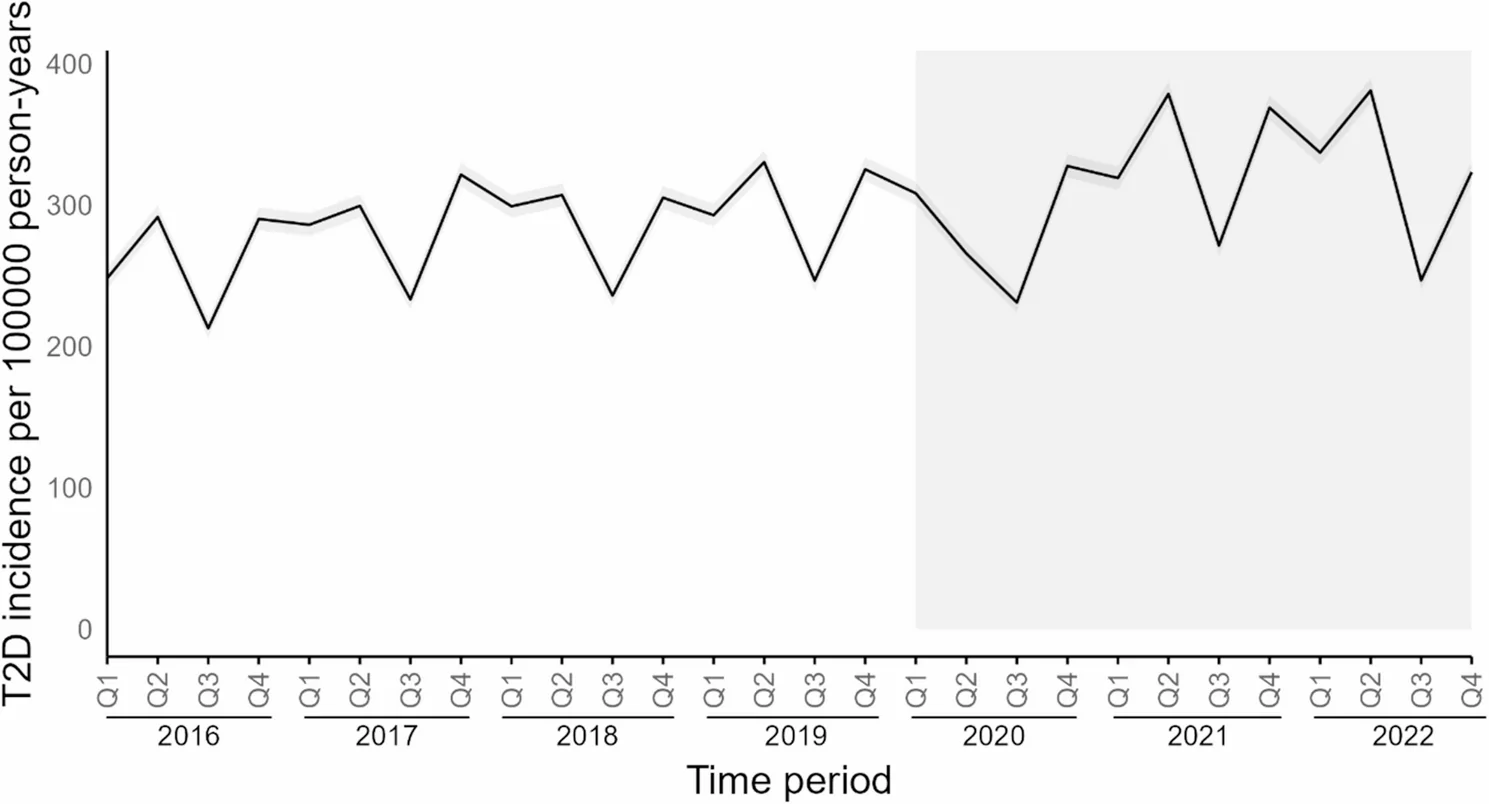
Trend in the quarterly incidence rate with 95% confidence intervals of type 2 diabetes per 100,000 person-years at risk between 2016–2022 in the adult population of Sweden. Pandemic period indicated by grey area. T2D = type 2 diabetes

**Fig. 3:**
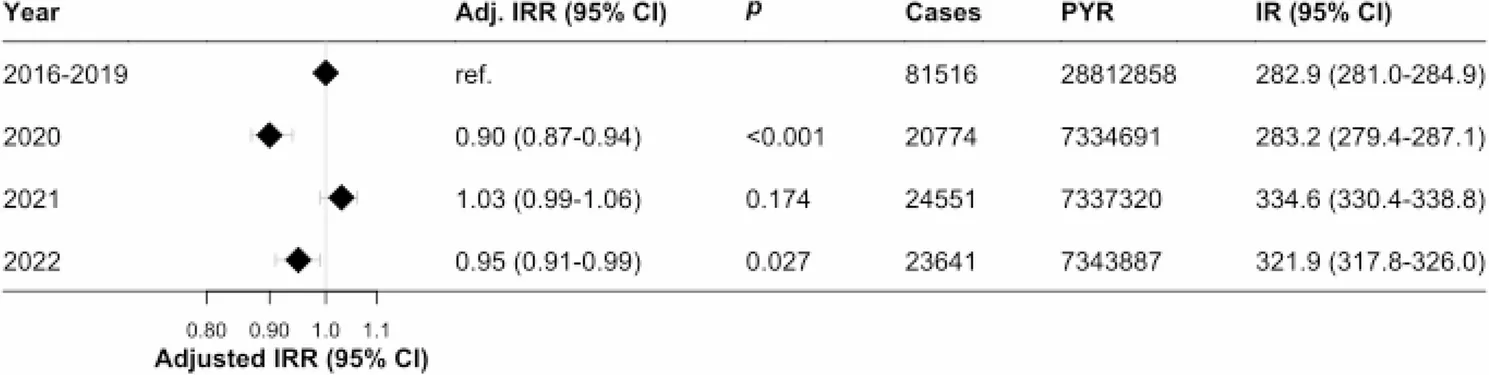
Adjusted incidence rate ratios (Adj. IRR) and unadjusted incidence rates (IR) with 95% confidence intervals (95% CI) of type 2 diabetes for each pandemic year (2020–2022) compared to the pre-pandemic period (2016–2019) in the adult population of Sweden. Adjusted IRRs were derived from Poisson regression model adjusted for age, sex, migration status, quarter, and the linear long-term annual trend. PYR = person-years at risk

In total, 8,672,344 adults with no record of diabetes before 2016 contributed 50,828,756 person-years at risk during 2016–2022. During the study period, 217,777 new type 2 diabetes cases were identified in the NDR, of which 150,482 cases fulfilled the study definition of incident type 2 diabetes. Cases were 59.5% male, 68.8% native-born, and the median age at onset was 62 (53–71) years (Table 1). There were 81,516 cases of new-onset type 2 diabetes during the pre-pandemic period 2016–2019 (IR 282.9 per 100,000 person-years, 95% CI 281.0, 284.9) and 68,966 cases during the pandemic period 2020–2022 (IR 313.3 per 100,000 person-years, 95% CI 310.9, 315.6). The overall IR per 100,000 person-years was 283.2 (95% CI 279.4, 287.1) in 2020, 334.6 (95% CI 330.4, 338.8) in 2021, and 321.9 (95% CI 317.8, 326.0) in 2022 (Figs. 2 and 3).

**Table 1:** Characteristics of type 2 diabetes cases stratified by pre-pandemic period and pandemic year

	Total	2016–2019	2020	2021	2022
*n*	150,482	81,516	20,774	24,551	23,641
Median age^a^	62 [53–71]	62 [53–70]	61 [52–70]	62 [53–71]	63 [53–72]
Age group^a^					
18–64 years	85,630 (56.9)	46,579 (57.1)	12,251 (59)	13,847 (56.4)	12,953 (54.8)
65 years and older	64,852 (43.1)	34,937 (42.9)	8523 (41)	10,704 (43.6)	10,688 (45.2)
Sex					
Female	60,870 (40.5)	32,558 (39.9)	8348 (40.2)	10,247 (41.7)	9717 (41.1)
Male	89,612 (59.5)	48,958 (60.1)	12,426 (59.8)	14,304 (58.3)	13,924 (58.9)
Migration status					
Native-born	103,571 (68.8)	57,259 (70.2)	13,795 (66.4)	16,468 (67.1)	16,049 (67.9)
Second-generation immigrant	10,503 (7)	5330 (6.5)	1498 (7.2)	1807 (7.4)	1868 (7.9)
Western origin	15,299 (10.2)	8402 (10.3)	2127 (10.2)	2479 (10.1)	2291 (9.7)
Non-Western origin	21,109 (14)	10,525 (12.9)	3354 (16.1)	3797 (15.5)	3433 (14.5)

Data are presented as median [25th -75th percentile] or *n* (%)

*Abbreviations*: *Western origin* first-generation immigrant with Western origin, *Non-Western origin* first-generation immigrant with non-Western origin

^a^ at onset of type 2 diabetes

The incidence of type 2 diabetes showed a positive underlying linear trend over the study period with a modelled average annual trend of 3.6% (IRR 1.036, 95% CI 1.028, 1.045) adjusted for age, sex, migration status, and quarter (see Supplementary Table 1). Type 2 diabetes incidence followed a consistent seasonal trend as the incidence decreased during the summer (third quarter), with spikes during the spring (second quarter) and fall (fourth quarter) (Fig. 2, Supplementary Table 1).

Over the total pandemic period 2020-2022, the observed type 2 diabetes incidence was 6% lower compared to the underlying linear trend (IRR 0.94, 95% CI 0.91, 0.97). In 2020, the incidence rate was 10% lower compared to the underlying linear trend (IRR 0.90, 95% CI 0.87, 0.94) (Fig. 3). In 2021, type 2 diabetes incidence returned to the expected underlying linear trend (IRR 1.03, 95% CI 0.99, 1.06). By 2022, type 2 diabetes incidence rate decreased compared to the underlying linear trend (IRR 0.95, 95% CI 0.91, 0.99). 

In the sensitivity analyses, 46,037 additional type 2 diabetes cases during the study period 2016-2022 were identified (196,519 cases in total). The results of the main analyses were robust under the alternative case definition. During 2020-2022 the incidence decreased by 4% (IRR 0.96, 95% CI 0.91, 1.00). In the respective pandemic years (2020-2022), the IRR were 0.92 (95% CI 0.88, 0.96) in 2020, 1.05 (95% CI 1.00, 1.11) in 2021, and 0.99 (95% CI 0.93, 1.05) in 2022 (Supplementary Figure 1).

### Differences in type 2 diabetes incidence changes according to age, sex, and migration status

**Fig. 4:**
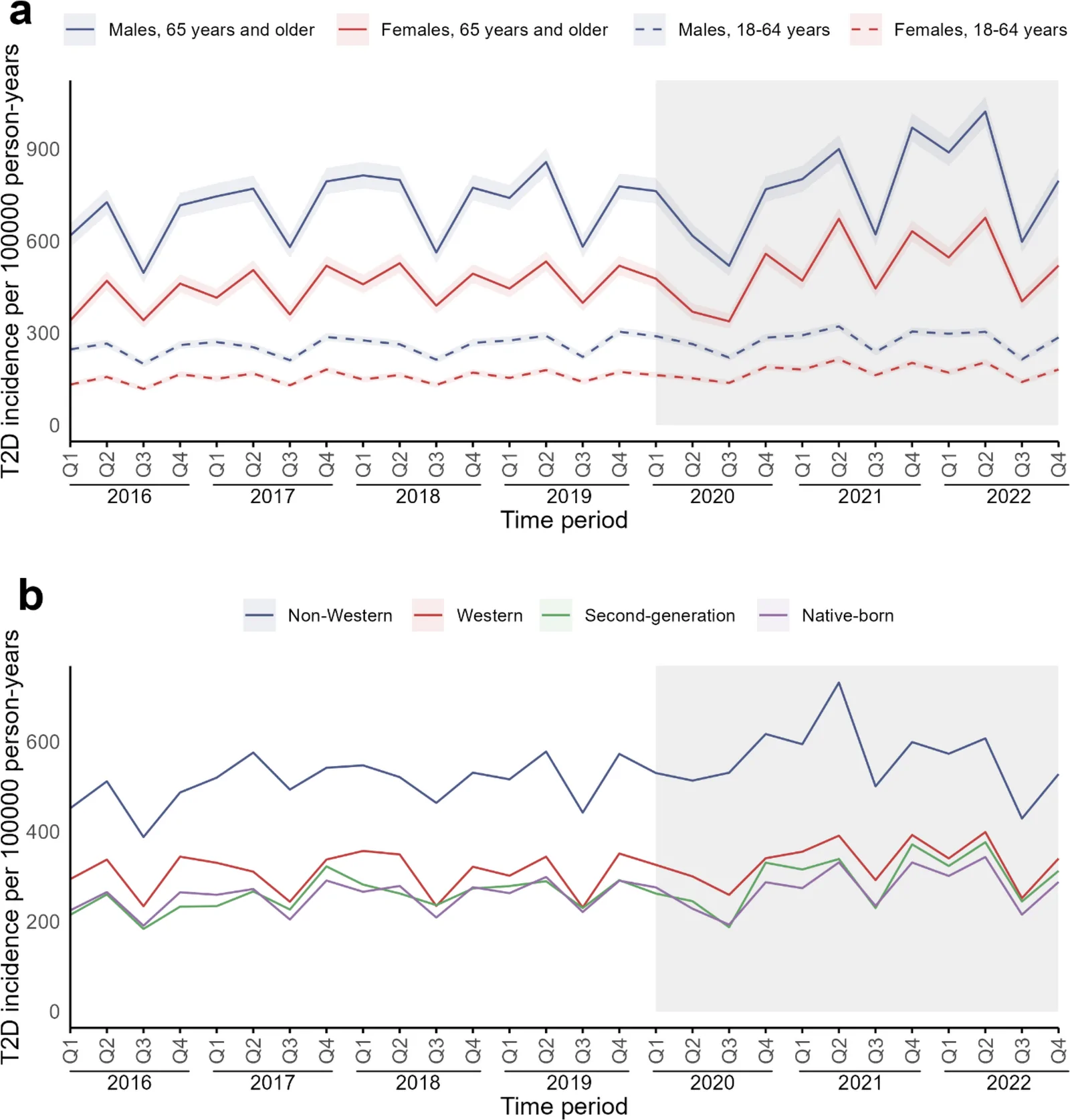
Trends in the quarterly incidence rates with 95% confidence intervals of type 2 diabetes per 100,000 person-years at risk between 2016-2022 in the adult population of Sweden stratified (**a**) by sex and age group and (**b**) by migration status. The incidence rates in (**b**) are age- and sex-standardized. Pandemic period indicated by grey area. Non-Western = first-generation immigrants with non-Western origin; Second-generation = second-generation immigrants; T2D = type 2 diabetes; Western = first-generation immigrants with Western origin

When stratified by sex and age, the incidence of type 2 diabetes was highest in males and individuals ≥65 years, compared to females and 18-64-year-olds, during 2016-2022 (Fig. 4a). Individuals born outside Sweden had higher incidence compared to second-generation immigrants or native-born, where first-generation migrants with non-Western origin had the highest overall incidence of new-onset type 2 diabetes 2016-2022 (Fig. 4b).

**Fig. 5:**
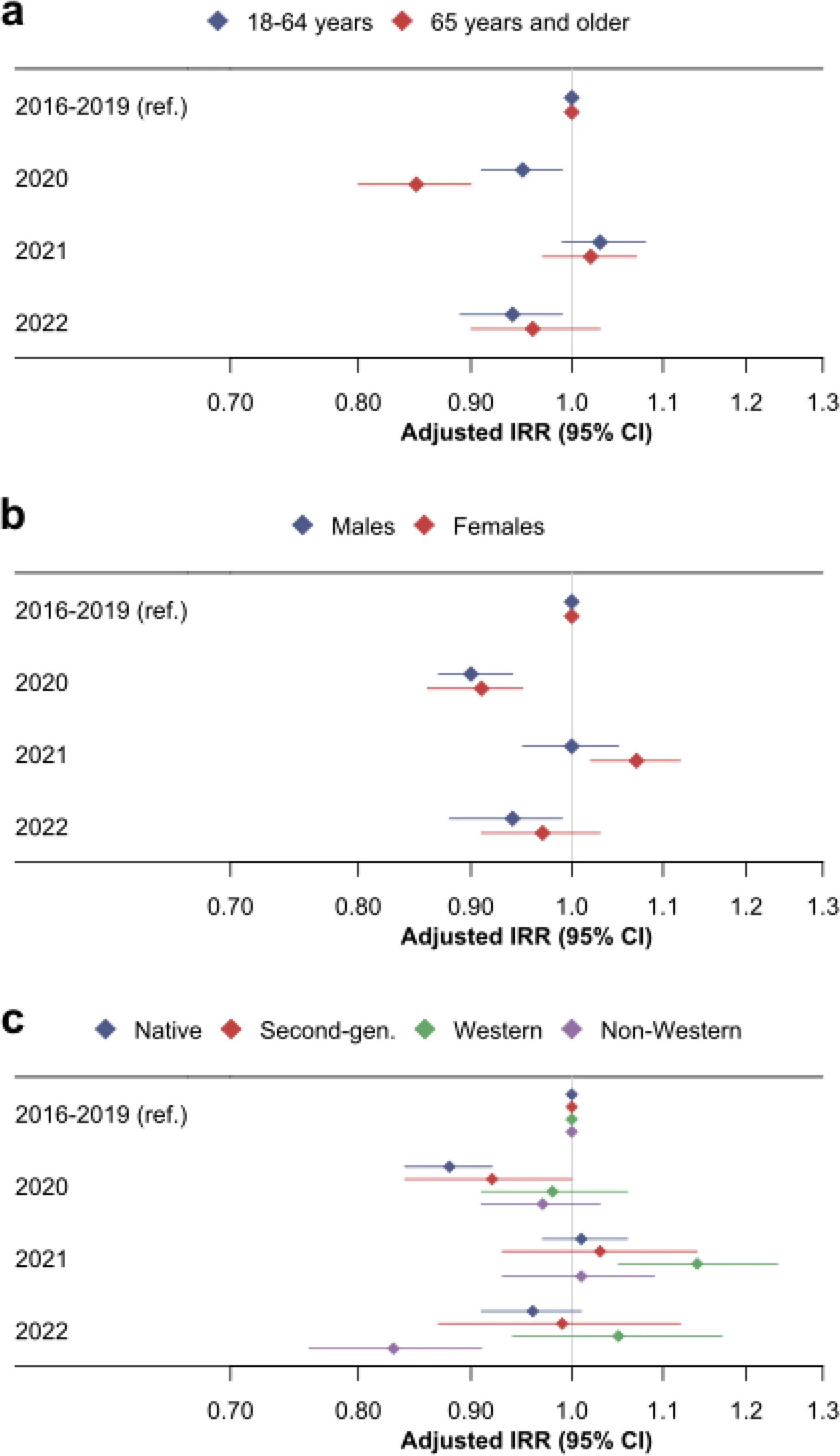
Adjusted incidence rate ratios (IRR) with 95% confidence intervals (95% CI) of type 2 diabetes in Swedish adults according to calendar year compared to the pre-pandemic period (2016-2019) stratified by (**a**) age group, (**b**) sex, and (**c**) migration status. Adjusted IRRs were derived from Poisson regression models adjusted for sex, age, migration status, quarter, and the linear long-term trend. Native = native-born; Non-Western = first-generation immigrants with non-Western origin; Second gen. = second-generation immigrants; Western = first-generation immigrants with Western origin

The underlying linear trend ranged between 1%-5% in all population groups (Supplementary Table 2). Figure 5 shows how the COVID-19 pandemic changed the underlying linear trend in type 2 diabetes incidence stratified by age (Fig. 5a), sex (Fig. 5b), and migration status (Fig. 5c). There was significant heterogeneity in the effect of the pandemic on type 2 diabetes incidence in relation to age, sex, and migration status (p for interaction <0.001) (Supplementary Table 3).

Compared to the underlying linear trend, the incidence during the total pandemic period was significantly lower in individuals 65 years and older (IRR 0.89, 95% CI 0.84, 0.94) but similar in 18-64-year-olds (IRR 0.98, 95% CI 0.94, 1.02). The incidence in 2020 decreased in individuals aged 18-64 years (IRR 0.95, 95% CI 0.91, 0.99) and in particular in 65 years and older (IRR 0.85, 95% CI 0.80, 0.90) but returned to the underlying linear trends in 2021 (Fig. 5a). By 2022, the incidence had returned to the underlying linear trend for ≥65-year-olds but not for 18-64-year-olds where decreased incidence rates were observed (IRR 0.94, 95% CI 0.89, 0.99).

Type 2 diabetes incidence in the total pandemic period was lower compared to the underlying linear trends in both females (IRR 0.95, 95% CI 0.91, 1.00) and males (IRR 0.93, 95% CI 0.89, 0.97). Compared to the underlying linear trends, the incidence decreased in 2020 in both males (IRR 0.90, 95% CI 0.87, 0.94) and females (IRR 0.91, 95% CI 0.86, 0.95) (Fig. 5b). In 2021, a marked increase versus the linear trend was observed in females (IRR 1.07, 95% CI 1.02, 1.12) but not in males (IRR 1.00, 95% CI 0.95, 1.05). By 2022, the incidence rate decreased in males (IRR 0.94, 95% CI 0.88, 0.99), while the incidence in females had returned to the underlying linear trend. 

The effect of the pandemic period on type 2 diabetes incidence was not as strong in the sensitivity analyses in 18-64-year-olds and in males as in the main analyses (Supplementary Figure 2a-b). Yet, heterogeneity in the effect of the pandemic on type 2 diabetes incidence was consistent with our data and remained significant in different age and sex groups (p for interaction <0.001).

Over the entire pandemic period, the type 2 diabetes incidence decreased in native-born (IRR 0.91, 95% CI 0.87, 0.95) but was coherent with the underlying linear trends in first-generation immigrants with Western and non-Western origin and in second-generation immigrants. The type 2 diabetes incidence decreased in 2020 in native-born (IRR 0.88, 95% CI 0.84, 0.92) (Fig. 5c). In 2021 and 2022, native-born had returned to the underlying linear trend. In 2021, the incidence significantly increased in first-generation immigrants with Western origin (IRR 1.14, 95% CI 1.05, 1.24) but then returned to the underlying linear trend in 2022. The incidence in first-generation immigrants with non-Western origin was similar to the underlying linear trend in 2021, but was significantly lower in 2022 (IRR 0.83, 95% CI 0.76, 0.91) (Fig. 5c).

The effect of the pandemic on type 2 diabetes incidence in relation to migration status was consistent in the sensitivity analysis (Supplementary Figure 2c), and the interaction analysis showed significant heterogeneity in the effect of the pandemic between the different groups (p for interaction <0.001).

## Discussion

To the best of our knowledge this study is the first to report type 2 diabetes incidence trends in the total adult population in Sweden across sex, age group, and migration status, comparing the COVID-19 pre-pandemic with the pandemic period. We show that the incidence of type 2 diabetes in Sweden decreased during the onset of the COVID-19 pandemic in 2020, compared to the underlying linear trend. The incidence returned to pre-pandemic estimates in 2021 before it decreased again below the underlying linear trend in 2022. Further, we found notable differences in relation to age group, sex, and migration status. The decline in type 2 diabetes incidence in 2020 was most evident in people ≥ 65 years of age and in native-born. Our data contribute important insights into disease burden trends among the total population as well as disparities within it during the pandemic.

A major strength of our study is the use of high-quality and extensive Swedish register data which allowed us to extract type 2 diabetes onset dates and model its incidence in the complete adult population using a combination of the national quality register NDR and a number of national public authority registers. The triangulation of data sources is particularly important because treatment guidelines of conditions such as heart and kidney failure have changed in recent years to also include glucose-lowering medication, extending its prescription beyond diabetes cases [33, 34]. Therefore, the commonly used approach to solely use data on drug prescription is not a reliable approach for identifying type 2 diabetes cases anymore [35].

The difficulty of identifying the date of type 2 diabetes onset should be considered when interpreting the results. As type 2 diabetes often is preceded by a subclinical pre-diabetic phase, the onset of type 2 diabetes is difficult to identify. Thus, the absence of evidence of type 2 diabetes cannot guarantee that an individual did not have unrecognised diabetes. In this study, we conditioned the onset date on a registration in the National Prescribed Drug Register or the National Patient Register to occur during the year of type 2 diabetes onset recorded in the NDR. Not all individuals with a registration in the NDR had a corresponding diabetes diagnosis or an anti-diabetic prescription in the same year. The National Patient Register covers inpatient and specialized outpatient care but not primary care, where many type 2 diabetes cases are diagnosed and managed [32]. NDR-registered cases treated exclusively in primary care will therefore lack a corresponding diagnosis in the National Patient Register, and if anti-diabetic medication was not dispensed in the same calendar year, no onset could be established. Neither register is a reliable sole indicator of new-onset type 2 diabetes, as noted above, glucose-lowering medication is increasingly prescribed for heart and kidney failure, and a diabetes diagnosis in the National Patient Register may relate to a complication of established type 2 diabetes rather than the initial diagnosis. According to the 2023 analysis by the NDR, coverage among adults with diabetes 50–79 years old was 85% compared to the National Prescribed Drug Register. Alternatively, among all individuals ever registered in the NDR and alive on 31 December 2023, 88% had a registered visit in diabetes care during 2022–2023 [28]. This appears consistent with our finding that 69% of all newly registered individuals with type 2 diabetes in the NDR had contacts with specialized diabetic care registered in the National Patient Register or a registered prescription of anti-diabetic medication when restricting to registrations in the same year. Taken together, roughly one third of new-onset type 2 diabetes cases in Sweden may not have been captured, and our incidence estimates are therefore more likely to be underestimates. As the NDR does not capture the month of diagnosis, the sensitivity analysis could not evaluate seasonal variation in new-onset type 2 diabetes. Nevertheless, the estimates for 2020, 2021, and 2022 relative to the pre-pandemic period were consistent with those of the primary analysis when case ascertainment was based solely on NDR registration. Theoretically, it can be shown that misclassification of disease outcome tends to dilute exposure-outcome associations if the misclassification is non-differential, that is independent of exposure status (here calendar period, age, sex, and migration status) [36].

Our study shows an increase in type 2 diabetes incidence prior to the COVID-19 pandemic in 2020 with a modelled average yearly trend of 3.6%, which is higher than estimates from the Global Burden of Disease Study 2019 of a 2% increase in type 2 diabetes incidence in high-income countries [37]. A steady increase in pre-pandemic type 2 diabetes incidence trend was further shown in studies from England and Italy [11, 12]. Our results showing type 2 diabetes incidence in Sweden to deviate negatively from the underlying linear trend in 2020 and return to the trend estimates in 2021 was corroborated by Barron et al. using whole population data from England [12]. They also found disparate impacts of the acute phase of the pandemic on type 2 diabetes incidence in different age groups which are consistent with our results. Incidence decreased to a larger extent in older persons in 2020, with the negative effect attenuated in younger individuals [12]. On the contrary, results from an Italian study showed a continuous increase of type 2 diabetes incidence already during the first pandemic year, as well as in 2021 and 2022 [11]. Similarly, a US study of adults with pre-diabetes found that monthly type 2 diabetes incidence increased following pandemic onset and persisted throughout 2021 [38]. The apparent inconsistency across studies likely reflects differences in study populations, healthcare context, and the degree to which the COVID-19 pandemic affected screening-driven case detection.

The decline in type 2 diabetes incidence during 2020 is potentially a result of changes in health-seeking behaviours. Indeed, the COVID-19 pandemic placed significant strain on Sweden’s healthcare system and the number of primary care visits for new or worsening health conditions declined during the first year of the pandemic [10]. The number of individuals registered in the NDR declined by 20% during the first half of 2020, compared to the same period 2019 [39]. By 2021, number of registrations were similar to that of 2019, and has since continued to increase annually [40]. The decline in registrations in 2020 may reflect disruptions in routine care, with healthcare professionals deprioritizing preventive diabetes care during the acute phase of the pandemic. Regular check-ups were also paused to reduce the risk of SARS-CoV-2 exposure, especially among older individuals, potentially leading to fewer diagnoses being made. Our results indicate a drop in type 2 diabetes incidence during the first pandemic year, particularly among the older age groups. This is in line with national surveys indicating that 12–34% of the population postponed or avoided seeking health care during this time, with the highest rates of avoidance among individuals 70–84 [41]. This could indicate that the strained health care and avoidance of health care seeking delayed diagnoses until conditions worsened. Indeed, a recent Turkish study revealed higher HbA_1C_ levels in patients with new-onset diabetes during the pandemic compared to the pre-pandemic period [42], indicating later detection of diabetes and prolonged hyperglycaemia during the pandemic times. Evidence from the United Kingdom suggest a 74%-88% reduction in diabetes care processes compared to the pre-pandemic period [43], alongside decreases in diabetes diagnoses and HbA_1C_ testing [44]. The largest reductions in health care checks were observed among older adults residing in socioeconomically deprived areas [43]. This suggests that missed or delayed diagnoses was not a single-nation event, but a pandemic consequence that affected populations also outside Sweden. Delayed detection and diagnosis of type 2 diabetes substantially increase the risk of microvascular complications, myocardial infarctions, and other disease-related complications [45, 46]. During health crises, such as pandemics, health care needs to ensure continued and high-quality care also for non-communicable diseases such as type 2 diabetes, with a special focus on tending to those most burdened by the disease [47]. Excess mortality during 2020 may also have influenced the incidence estimates if those who died had a higher-than-average risk of type 2 diabetes, thereby depleting the population at risk of its most susceptible members. A similar competing-risk mechanism has been proposed to explain the decline in hospital admissions for acute cardiovascular disease during the pandemic [48]. Although the relative contributions of excess mortality and delayed diagnosis cannot be disentangled with certainty, depletion of susceptible individuals would result in permanently lost cases rather than a later rebound in incidence, and would be expected to be most pronounced in older age groups. The rebound in incidence observed during 2021 among individuals aged 65 years or older therefore argues against depletion as the sole explanation. The more pronounced rebound in the sensitivity analysis based on NDR registration alone, likely capturing diagnoses registered in primary care, further supports delayed diagnosis as a plausible explanation for the decrease in incidence during 2020.

Our results further indicate that following a drop among both males and females in type 2 diabetes incidence in 2020, there was an increase in 2021 only among females, while incidence among males returned to the underlying trend. This suggests that some cases among females that developed clinical symptoms of type 2 diabetes in 2020 may have delayed seeking care during the acute phase of the COVID-19 pandemic. These cases were then diagnosed to some extent in the subsequent years when standard diabetes care was reinstated and public health recommendations were lifted. Additionally, the availability of SARS-CoV-2 vaccines in 2021, may have contributed to reduced barriers to seeking care. Vaccination coverage was higher among females [49], who also tend to utilize primary care services more frequently than males [50, 51]. Taken together, these factors may, at least in part, account for the observed sex differences in type 2 diabetes incidence. An alternative, but possibly related, hypothesis is the pandemic effects on mental health and lifestyle factors. The association between psychosocial factors and type 2 diabetes have been reported to be stronger in females compared to males [52]. At the same time, findings indicate females exhibiting stress and anxiety to a higher extent relative to males during the pandemic [53]. Stressors such as increased job insecurities, childcaring responsibilities [54, 55], and domestic violence [54], was to a higher degree experienced in females compared to males. This holds especially true for females in an already vulnerable socioeconomic situation [54]. In addition, Sweden adopted a relatively non-restrictive public health response which relied on interventions primarily based on public health recommendations, such as working from home. A report from the Swedish Public Health Agency on how the pandemic affected the health in the population, reported decreased physical activity and vegetable consumption, and an increased consumption of snacks and sweets [56]. The negative effect of a sedentary lifestyle on psychological well-being has been reported to be greater in females [57]. As the onset of type 2 diabetes is gradual and preceded by a pre-diabetic phase, future research is necessary to explore how incidence trends continued to evolve further beyond the pandemic years. As incidence decreased in males 2022, females returned to their underlying trend estimates. Delayed or missed health care seeking among males furthermore raises concern regarding sex-differences in post-pandemic disease-related complications.

Our findings suggest that the pandemic affected type 2 diabetes incidence differently across migration-status groups. In 2020, incidence fell below underlying trends among native-born individuals and second-generation immigrants, whereas it followed the underlying trend for first-generation immigrants. One possible explanation is differential pathways to diagnosis. Type 2 diabetes is often detected incidentally during routine check-ups, health screenings, or follow-up visits for other health conditions. Individuals who have experienced discrimination are less likely to seek health care [58, 59], and migrants in Europe more frequently use emergency services for low-acuity conditions, suggesting barriers to primary and secondary care [60]. By contrast, native-born individuals are more likely to participate in routine health screening programs [61], increasing opportunities for incidental diabetes detection. The marked reduction in non-acute healthcare utilization during the first pandemic year may therefore have disproportionally reduced opportunities for diagnosis among native-born individuals and second-generation immigrants, consistent with the observed decline in incidence. In contrast, if first-generation immigrants have lower levels of preventive healthcare contact at baseline, diabetes diagnoses in this group may more often occur following symptomatic presentation, which was less affected by pandemic-related disruptions. Consequently, interruptions to routine screening may have had a smaller impact in a population that was already comparatively under-screened.

A similar pattern has been reported outside Sweden. In the United States, type 2 diabetes incidence among pre-diabetic Hispanic adults increased 51% more than among White adults following pandemic onset, with differences persisting through 2021, whereas no significant changes were observed in other groups [38]. Although healthcare systems and population contexts differ, these findings suggest that pandemic-related healthcare contacts, potentially driven by disproportionate COVID-19 morbidity and hospitalization in minority populations, may have sustained or increased diabetes detection in groups with limited access to routine preventive care.

Some studies have suggested that SARS-CoV-2 infections may directly contribute to the development of type 2 diabetes. However, in our study [17], the observed association between SARS-CoV-2 infection and incident type 2 diabetes may reflect increased case detection among individuals hospitalized with COVID-19 rather than a direct effect of the infection itself. Hence, other explanations, such as health-seeking patterns, are more likely to explain the findings of this study.

## Conclusions

This study highlights the impact of the COVID-19 pandemic on type 2 diabetes incidence in Sweden and the disparate effects across groups. Future studies should assess the extent to which missed or delayed type 2 diabetes diagnoses during the first year of the pandemic affected disease progression and the risk of adverse outcomes. Furthermore, a better understanding of how the pandemic affected type 2 diabetes risk factors and health-seeking behaviours is needed.

## Supplementary Information

Additional file 1: Supplementary Methods 1: Description of the register data; National Diabetes Register (NDR), National Patient Register (NPR) and National Prescribed Drug Register, providing information on diabetes diagnosis. Supplementary Methods 2: Calculation of age and sex specific weights. Supplementary Table 1: Estimates of the main Poisson regression model. Supplementary Table 2: Underlying linear trends of type 2 diabetes incidence from Poisson regression models stratified by sex, age group, and migration status. Supplementary Table 3: Coefficients from interaction analysis, pandemic impact on type 2 diabetes incidence by sex, age group, and migration status. Supplementary Figure 1: Results from sensitivity analysis, incidence of type 2 diabetes in 2020, 2021, and 2022 compared to 2016-2019 in the total population. Supplementary Figure 2a-c: Results from sensitivity analysis, incidence of type 2 diabetes in 2020, 2021, and 2022 compared to 2016-2019 by (a) age group, (b) sex, and (c) migration status.

## Data Availability

The individual-level data used in this paper was retrieved from a variety of Swedish registers kept by public agencies such as Statistics Sweden and the National Board of Health and Welfare, and the Centre of Registers Västra Götaland. The data are classified and not publicly available due to data privacy laws. Access to the data requires approval from the relevant register holders following ethical review.

## Abbreviations

ATC: Anatomical Therapeutic Chemical Classification System
IR: Incidence Rate
IRR: Incidence Rate Ratio
LRT: Likelihood Ratio Test
NDR: Swedish National Diabetes Register
SARS-CoV-2: Severe Acute Respiratory Syndrome Coronavirus 2
SWECOV: Swedish Register-based Research Program on COVID-19
