# Supplementary material for: Disparities in type 2 diabetes incidence trends during the COVID-19 pandemic in Sweden: a register-based population study
Source: BMC Public Health. 2026 Oct 28;26:3100. doi: 10.1186/s12889-026-30127-z (PMC13715193; doi:10.1186/s12889-026-30127-z)
Supplement: Supplementary file 1 [file 12889_2026_30127_MOESM1_ESM.docx]

**Supplementary material**

**Disparities in the Type 2 Diabetes Incidence Changes during the COVID-19 Pandemic in Sweden: a Register-Based Population study**

J. Andreasson, D. Dietler, J. Björk & L. Bennet.

| **Supplementary Methods 1** | Description of the register data; National Diabetes Register (NDR), National Patient Register (NPR) and National Prescribed Drug Register, providing information on diabetes diagnoses |
| --- | --- |
| **Supplementary Methods 2** | Calculation of age and sex specific weights |
| **Supplementary Table 1** | Estimates of the main Poisson regression model |
| **Supplementary Table 2** | Underlying linear trends of type 2 diabetes incidence from Poisson regression models stratified by sex, age group, and migration status |
| **Supplementary Table 3** | Coefficients from interaction analysis, pandemic impact on type 2 diabetes incidence by sex, age group, and migration status |
| **Supplementary Figure 1** | Results from sensitivity analysis, incidence of type 2 diabetes in 2020, 2021, and 2022 compared to 2016-2019 in the total population |
| **Supplementary Figure 2a-c** | Results from sensitivity analysis, incidence of type 2 diabetes in 2020, 2021, and 2022 compared to 2016-2019 by (a) age group, (b) sex, and (c) migration status |

***Supplementary Methods 1.*** *Description of register data providing information on diabetes diagnoses.*

The Swedish National Diabetes Register (NDR) is a nationwide quality register. According to the 2023 analysis by the NDR, coverage among adults with diabetes 50-79 years old was 85% compared to the National Prescribed Drug Register. Alternatively, among all individuals ever registered in the NDR and alive on 31 December 2023, 88% had a registered visits in diabetes care during 2022-2023 NDR collects data from health care on children and adults diagnosed with type 1 diabetes (including latent autoimmune diabetes in adults), type 2 diabetes, and other specified forms of diabetes, excluding gestational diabetes.

The National Patient Register (NPR) and the National Prescribed Drug Register (NPDR) are both administered by the National Board of Health and Welfare. The NPR underpins official statistics on diseases and treatments in Swedish specialized care. This health data register is governed by Swedish legislation that defines the information to be included and mandates reporting by health care providers The NPDR includes data on all prescribed drugs dispensed at Swedish pharmacies and forms the basis for official pharmaceutical statistics, with a particular emphasis on patient safety All pharmacy retailers are required to report dispensed prescriptions and related dispensing information to the Swedish eHealth Agency, which subsequently transfers these data to the National Board of Health and Welfare.

**Supplementary Methods 2**. Calculation of age- and sex-specific weights.

Calculation of age- and sex-specific weights (*W_i_)* was based on the proportion of individuals in each sex- and 5-year age-group relative to the total population.

$$W_{i}={N_{i}}/N$$

where *N_i_* is the number of individuals in sex-age group *i* and *N* is the total population. For each sex-age group, the incidence rate (*I_iq_)* was calculated for each quarter (*q*), and its standard error was estimated as:

$${SE}_{iq}=\sqrt{{n_{iq}}/{p_{iq}^{2}}}$$

where *n_iq_* is the number of cases, and *p_iq_* is the number of person-years at risk. The standardized incidence rate (*I_iq,s_*) was obtained by applying the sex-age-specific weights:

$$I_{iq,s}=I_{iq}\times W_{i}$$

The standard error of the weighted sum was calculated as:

$${SE}_{iq,s}= \sqrt{\sum_{i} ({{(W}_{i})}^{2}\times{{SE}_{iq}}^{2})}$$

The 95% CIs were estimated using the standard errors of the weighted sums:

$${CI}_{iq,s}=I_{iq,s} \pm1.96\times{SE}_{iq,s}$$

**Supplementary Table 1**. Estimates of the Poisson regression model estimating the pandemic impact on type 2 diabetes incidence in the total population with p-value and lower and upper 95% CIs. Second-gen. = second-generation immigrant; Western = first-generation immigrant with Western origin; Non-Western = first-generation immigrant with non-Western origin.

|  | **Estimate** | **Lower 95% CI** | **Upper 95% CI** | ***p*-value** |
| --- | --- | --- | --- | --- |
| Intercept | 0.000 | 0.000 | 0.000 | <0.001 |
| **Pandemic year** |  |  |  |  |
| 2020 | 0.905 | 0.874 | 0.937 | <0.001 |
| 2021 | 1.026 | 0.989 | 1.065 | 0.174 |
| 2022 | 0.949 | 0.907 | 0.994 | 0.027 |
| **Age group** |  |  |  |  |
| 25-29 years | 3.982 | 3.479 | 4.557 | <0.001 |
| 30-34 years | 7.269 | 6.379 | 8.283 | <0.001 |
| 35-39 years | 12.904 | 11.336 | 14.688 | <0.001 |
| 40-44 years | 22.466 | 19.751 | 25.554 | <0.001 |
| 45-49 years | 36.313 | 31.980 | 41.233 | <0.001 |
| 50-54 years | 53.228 | 46.963 | 60.329 | <0.001 |
| 55-59 years | 75.183 | 66.377 | 85.157 | <0.001 |
| 60-64 years | 92.109 | 81.314 | 104.336 | <0.001 |
| 65-69 years | 103.659 | 91.471 | 117.471 | <0.001 |
| 70-74 years | 106.958 | 94.332 | 121.274 | <0.001 |
| 75-79 years | 99.148 | 87.161 | 112.784 | <0.001 |
| 80-84 years | 72.268 | 63.447 | 82.316 | <0.001 |
| 85 years and older | 30.370 | 26.532 | 34.764 | <0.001 |
| **Male** | 1.602 | 1.575 | 1.630 | <0.001 |
| **Migration status** |  |  |  |  |
| Second-gen. | 1.071 | 1.047 | 1.097 | <0.001 |
| Western | 1.206 | 1.180 | 1.233 | <0.001 |
| Non-Western | 2.273 | 2.219 | 2.330 | <0.001 |
| **Yearly trend** | 1.036 | 1.028 | 1.045 | <0.001 |
| **Quarter** |  |  |  |  |
| 2 | 1.076 | 1.050 | 1.103 | <0.001 |
| 3 | 0.799 | 0.782 | 0.818 | <0.001 |
| 4 | 1.077 | 1.053 | 1.102 | <0.001 |

**Supplementary Table 2**. Underlying linear trends with lower and upper 95% CI and p-value of type 2 diabetes incidence over the study period 2016-2022 from Poisson regression models stratified by (1) sex, (2) age group, and (3) migration status, adjusted for age, sex, migration status. Second-gen. = second-generation immigrant; Western = first-generation immigrant with Western origin; Non-Western = first-generation immigrant with non-Western origin.

| **Group** | **Estimate** | **Lower 95% CI** | **Upper 95% CI** | ***p*-value** |
| --- | --- | --- | --- | --- |
| **Sex** |  |  |  |  |
| Male | 1.036 | 1.026 | 1.047 | <0.001 |
| Female | 1.037 | 1.025 | 1.048 | <0.001 |
| **Age group** |  |  |  |  |
| 18-64 years | 1.028 | 1.017 | 1.039 | <0.001 |
| 65 years and older | 1.048 | 1.035 | 1.061 | <0.001 |
| **Migration status** |  |  |  |  |
| Native-born | 1.038 | 1.028 | 1.049 | <0.001 |
| Second-gen. | 1.047 | 1.020 | 1.074 | 0.001 |
| Western | 1.010 | 0.990 | 1.028 | 0.544 |
| Non-Western | 1.047 | 1.028 | 1.066 | <0.001 |

**Supplementary Table 3**. Coefficients with lower and upper 95% CI and p-value from interaction analysis analyzing the pandemic impact on type 2 diabetes incidence in (1) males, compared to females, (2) individuals 65 years and older, compared to individuals 18-64 years, and (3) second-generation immigrants and first-generation immigrants with non-Western and Western origin compared to native-born, during the same pandemic year. Second-gen. = second-generation immigrant; Western = first-generation immigrant with Western origin; Non-Western = first-generation immigrant with non-Western origin.

| **Interaction term** | **Estimate** | **Lower 95% CI** | **Upper 95% CI** | ***p*-value** |
| --- | --- | --- | --- | --- |
| **Sex** |  |  |  |  |
| 2020:Male | 0.990 | 0.935 | 1.050 | 0.746 |
| 2021:Male | 0.930 | 0.886 | 0.977 | 0.004 |
| 2022:Male | 0.957 | 0.905 | 1.012 | 0.126 |
| **Age group** |  |  |  |  |
| 2020:65 years and older | 0.931 | 0.877 | 0.989 | 0.020 |
| 2021:65 years and older | 1.041 | 0.993 | 1.091 | 0.095 |
| 2022:65 years and older | 1.105 | 1.049 | 1.165 | <0.001 |
| **Migration status** |  |  |  |  |
| 2020:Second-gen. | 1.061 | 0.988 | 1.141 | 0.105 |
| 2021:Second-gen. | 1.035 | 0.973 | 1.100 | 0.281 |
| 2022:Second-gen. | 1.058 | 0.991 | 1.130 | 0.093 |
| 2020:Western | 1.036 | 0.965 | 1.112 | 0.334 |
| 2021:Western | 1.008 | 0.951 | 1.068 | 0.783 |
| 2022:Western | 0.950 | 0.892 | 1.012 | 0.112 |
| 2020:Non-Western | 1.122 | 1.041 | 1.210 | 0.003 |
| 2021:Non-Western | 1.013 | 0.944 | 1.087 | 0.725 |
| 2022:Non-Western | 0.894 | 0.837 | 0.955 | 0.001 |

**Supplementary Figure 1**. Sensitivity analysis of adjusted incidence rate ratio (Adj. IRR) and unadjusted incidence rate (IR) with 95% CI of type 2 diabetes in Swedish adults according to calendar year in the total population using only year of onset in the NDR to define type 2 diabetes case date. Incidence rate ratios were derived from Poisson regression model adjusted for age, sex, and migration status.

**Supplementary Figure 2.** Sensitivity analysis of adjusted incidence rate ratio (IRR) with 95% CI of type 2 diabetes in Swedish adults according to calendar year by (a) age group, (b) sex, and (c) migration status using only year of onset in the NDR to define type 2 diabetes case date. Incidence rate ratios were derived from Poisson regression models adjusted for sex, age, and migration status.
